# A New Experimental Method for *in Situ* Corrosion Monitoring Under Alternate Wet-Dry Conditions

**DOI:** 10.3390/s91210400

**Published:** 2009-12-21

**Authors:** Xinxin Fu, Junhua Dong, Enhou Han, Wei Ke

**Affiliations:** State Key Laboratory for Corrosion and Protection of Metals, Institute of Metal Research, Chinese Academy of Science, Shenyang 110015, China; E-Mails: xxfu@imr.ac.cn (X.F.); ehhan@imr.ac.cn (E.H.); kewei@imr.ac.cn (W.K.)

**Keywords:** alternate wet-dry condition, mild steel, electrochemical impedance spectroscopy (EIS), electrolyte film, electrical balance

## Abstract

A new experimental method was applied in *in situ* corrosion monitoring of mild steel Q235 under alternate wet-dry conditions. The thickness of the electrolyte film during the wet cycle was monitored by a high-precision balance with a sensibility of 0.1 mg. At the same time, an electrochemical impedance technique was employed to study the effect of film thickness on corrosion rates. Experimental results showed that there was a critical electrolyte film condition for which the corrosion rate reached a maximum during wet-dry cycles. For the substrate, the critical condition could be described by a film thickness of about 17 μm. For the rusted specimen, the critical condition could be described by an electrolyte amount of about 0.038 g, which is equivalent to a film thickness of 38 μm. This monitoring system was very useful for studying atmospheric corrosion of metals covered by corrosion products.

## 1. Introduction

Atmospheric corrosion is an electrochemical process which occurs under thin dilute electrolyte. According to Tomashov, the atmospheric corrosion rate of metals depends on the thickness of electrolyte film on the surface [1]. This has attracted interests from many researchers. Mansfeld *et al.* [2] monitored the corrosion of metals covered with an electrolyte layer during drying using an atmospheric corrosion monitor (ACM), and found that the corrosion rate abruptly increased immediately before the surface dried out. Stratmann *et al.* [3] reported a set of equipments for monitoring the electrolyte layer thickness by measuring the potential difference between the electrode surface and a probe needle. Their study on oxygen reduction during dying of a platinum surface covered with a neutral solution layer indicated a maximum corrosion rate at a thickness of several tens of microns [4]. Nishikata *et al.* [5] reported that the corrosion rate of copper covered with an acidic sodium sulfate solution film showed a maximum at the thickness of several tens of micrometers, which was measured with a similar method. The experimental results reported by Yamashita *et al.* [6] on the layer thickness dependencies of the corrosion rate of low alloy steel indicated that the corrosion rate reached a maximum when the layer thickness is about 10 μm.

As the most widely used structural material and the largest source of losses caused by corrosion, the study of atmospheric corrosion of steels is of great significance. However, with low corrosion resistance, the surface of carbon steels can be severely rusted after numerous wet-dry cycles, which greatly influences the accuracy of traditional film thickness monitoring methods.

Electrochemical techniques have been widely used in atmospheric corrosion studies. In traditional electrochemical measurements a tri-electrode system with a Luggin capillary is often employed, but the Luggin capillary causes the properties of the measured surface to change abruptly, thus producing great errors. Zhang and Lyon [7] measured the polarization curves of metal covered with a thin electrolyte layer with an improved tri-electrode system. In their experiments, a working electrode with a very small working area was employed to reduce the ohmic drop between the working electrode and the Luggin capillary. In another study on atmospheric corrosion of iron, they used a bi-electrode system to avoid the effect of the reference electrode [8]. In studying atmospheric corrosion Nishikata *et al.* [2,9-12] used both bi-electrode and tri-electrode systems with very small distances between the electrodes to reduce the ohmic drop. Shi *et al.* [13] used a self-designed tri-electrode system, which consisted of a rod working electrode and a platinum circle counter electrode, to study the corrosion of 2024-T3 aluminum alloy in simulated acid rain. Li *et al.* [14] used chip-shaped bi-electrodes to study the evolution of EIS of carbon steel in a wet-dry process.

In the present paper, a new system for *in situ* measurement of the thickness of electrolyte films during simulated wet-dry atmospheric corrosion cycles is introduced. The electrochemical impedance spectroscopy (EIS) technique is employed with this method to monitor the corrosion process of low alloyed steels and study the difference between the corrosion characteristics of substrate and rusted specimens.

## 2. Experimental Section

### 2.1. Electrochemical Cell

**Figure 1.**
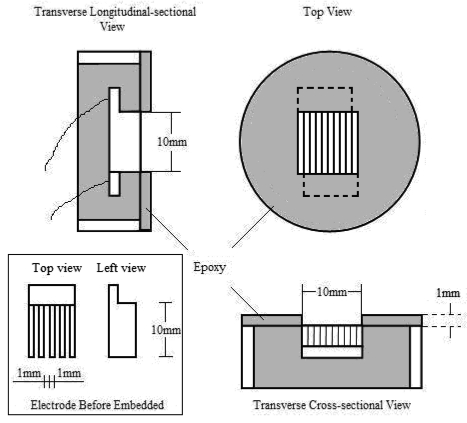
Schematic diagrams of the two-electrode cell used for EIS measurement.

In the present study, a two electrode cell arrangement was used for the EIS measurements to reduce errors caused by ohmic drop. The schematic diagram of the cell is shown in Figure 1. Test materials (mild steel Q235, whose chemical compositions are shown in Table 1) were cut into pairs of identical comb-shaped plates, then polished down with #2000 SiC papers, and ultrasonically cleaned with acetone. Then the pair of plates was embedded in epoxy resin with the comb fingers crossing each other. Before the experiment, the working surface of the cell was polished with SiC papers and diamond polishing paste (particle size 1 μm). A 1 mm height epoxy barrier was set around the electrode to ensure the constancy of the water film area during the wet period.

### 2.2. Electrolyte Thickness Monitoring

**Figure 2.**
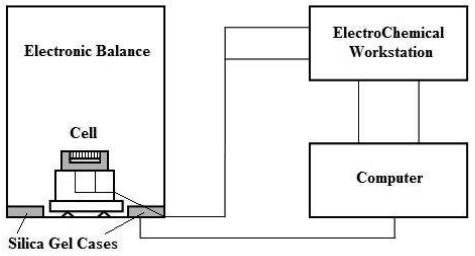
The EIS measuring system device.

As shown in Figure 2, the cell was fixed in a PMMA pedestal, connected to the electrochemical workstation by wires that were welded on the plates, and then positioned on the stage of a high-precision electronic balance (Sartorius, 0.1 mg) for *in situ* weight measurement. The connecting wires were fixed tightly so that the weight monitoring process won't be affected. Desiccant (silica gel) cases were put in the four corners of the balance in order to maintain a relatively constant humidity inside the balance. During the wet-dry cycles, readings of the balance were automatically recorded every 30 s by software. The room temperature was kept at 25 °C.

### 2.3. EIS Monitoring

EIS monitoring was conducted over the frequency range of 10 mHz to 100 kHz using a potentiostat (M273A) and an amplifier (5210E) which were controlled by a personal computer through the Powersuite interface.

### 2.4. Wet-Dry Exposure

The wet-dry exposure experiment was carried out for 60 cycles. At the beginning of the first cycle of the wet-dry alternation, certain volume (around 50 μL) of 0.05 M sodium chloride solution was added to the surface of the specimen to achieve a thin electrolyte film. The EIS measurement was carried out once every 15 min for 8 times during the evaporation of the electrolyte, until the surface was completely dried. From the second cycle, distilled water of same volume was added to the surface instead of sodium chloride solution. In this experiment, a regular wash after each cycle was skipped in order to avoid damage to the rust and to keep the amount of chloride ions in the electrolyte film unchanged in each cycle [15,16].

## 3. Results and Discussion

The weight of the electrolyte film (*W_e_*) at any given time can be calculated from the monitored weight of the specimen (*W_s_*) at this time minus the weight of the completely dried specimen (*W_d_*):

(1) $$W_{e}=W_{s}-W_{d}$$

Thus, electrolyte thickness *X* can be calculated by the following formula:

(2) $$X=\frac{W_{e}}{\rho S}$$

where ρ and S stand for the density and area of the electrolyte film respectively. In this experiment, ρ is the density of water, and S is 1 cm^2^.

**Figure 3.**
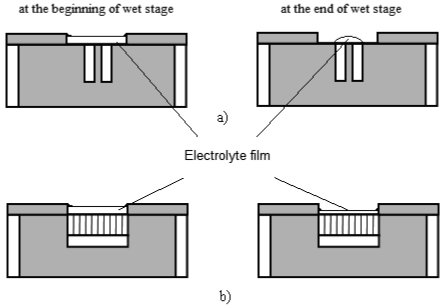
Comparison of different electrodes during the wet stage. (a) chip-shaped electrode. (b) comb-shaped electrode.

Electrodes used in electrochemical experiments are mostly chip-shaped, rod-shaped or square-shaped with small surface areas. The main difference between these electrodes and comb-shaped electrodes which were used in this experiment is the electrode area proportion, that is, the proportion of the area of electrode surface in the area covered by electrolyte film. Figures 3a,b illustrate the effect of different electrode area proportionss on the measurement of the electrolyte film thickness during wet-dry cycles. To avoid acutely uneven current distributing over the electrolyte film, the area of working surface (*i.e.*, surface covered by electrolyte film) cannot be too small. Therefore, as is shown in Figure 3a, the electrode area proportion of normal chip electrodes is quite small [14]. However in this experiment, the accurate calculation of electrolyte thickness with Equation (2) demands a constant electrolyte film area. Due to the different wetting condition of water solution on metal and epoxy, the electrolyte film tends to shrink to metal surface and form an electrolyte drop instead of film at the end of a wet stage, which causes the area of the film to change continuously, especially when the critical wet/dry condition is reached.

In this situation, the area of electrolyte film (S) becomes non-constant. This will cause great errors in the calculation of the thickness using Equation (2). Furthermore, during the corrosion reaction, the diffusion of oxygen is easier at the edge of the drop where the electrolyte is thinner, thus the reduction of oxygen occurs mostly at the edge, and things are reversed in the center of the drop. Thus the edge of drop turns into the cathode region, while the center of the drop turns into the anode region. This can cause uneven current distribution. As long as the above factors were concerned, in this experiment comb-shaped electrodes were employed (Figure 3b), which enlarged the electrode area proportions, and hence ensured the accuracy of the calculation and improved the current distribution situation.

Since both ρ and S can be regarded as constants in practice, the accuracy of X depends on the accuracy of *W_e_*. When the precision of the balance is 0.1 mg, this method enabled us to monitor the thickness of the electrolyte film with a sensibility of 1 μm.

**Figure 4.**
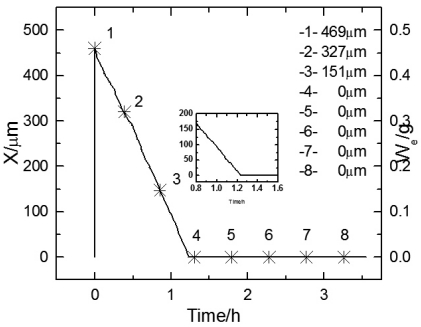
Plots of electrolyte film thickness X *vs.* time during 1^st^ cycle (substrate).

Figure 4 shows the thickness change of electrolyte film over time during the first wet-dry cycle. The electrode was the substrate in this cycle. The right Y axis shows the monitored weight of the electrolyte layer, and the left Y axis shows the thickness calculated from the weight. It can be seen that with the increase of time, the thickness curves first drop down with a steady slope till zero, then remain at zero. This indicates the feasibility of the weight monitoring method.

**Figure 5.**
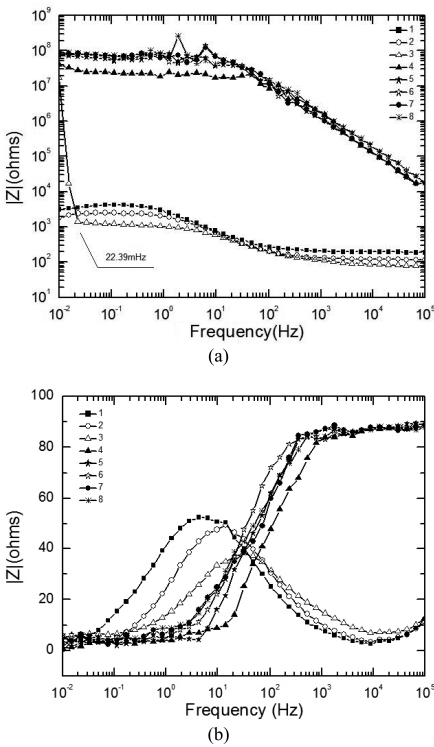
EIS results of 1^st^ cycle. (a) Bode diagram (|Z|). (b) Bode diagram (Phase of Z).

Figures 5a, b display the experimental Bode diagram of all eight measurements in the first cycle. After this cycle, there was little corrosion product formed, and the surface remained substrate. The starting time of the 1^st^ ∼8^th^ EIS measurement in Figure 5 are represented by the data points 1∼8 in Figure 4. By comparing the first three measurements in Figure 5a, it can be observed that with the evaporation of the electrolyte film, the impedances at both high and low frequency drop, which indicates that the corrosion rate is accelerating. However, when the thickness of the film keeps decreasing, the impedance shows an abrupt rise. This indicates that there exists a certain value of critical film thickness, around which the corrosion rate reaches a maximum. Once the thickness is beneath this range, the corrosion rate declines rapidly. This phenomenon is caused by several factors due to the atmospheric corrosion reaction mechanism. The evaporation of electrolyte film has two effects: an increase in the electrolyte concentration, and a decrease in the film thickness. The former effect results in a decrease of the solution resistance (R_s_), and thus accelerates the corrosion rate, while the latter acts in a more complicated way. On one hand, it causes the cross-sectional area of the electrolyte film to decrease, thus improves the solution resistance in the direction parallel to the interface plane. On the other hand, it also influences on both the oxygen gas transport rate through the electrolyte film and the solubility of corrosion products. The oxygen gas transport rate controls the rates of cathodic reaction of metallic corrosion, and the solubility of corrosion products affects the rate of the anodic process. The thinner the film is, the faster the oxygen transports to the metal surface, and the higher the cathodic reaction rate is; while the less the solubility of corrosion products is, and the lower the anodic reaction rate is. In addition, a change in the concentration of existing anions (Cl^-^, SO_4_^-^, CO_3_^-^, *etc.*) and probably a change in the pH of electrolyte occur with the change of thickness of the electrolyte film [5]. This conforms to the theory put forward by Tomashov [1].

An abrupt impedance rise took place at 22.39 mHz during the third measurement. The thickness of electrolyte film at this time could be approximated as 17 μm from the data record time. By comparing the first three measurements in Figure 5b, the phase angle exhibits a shift in the direction of higher frequency and also a drop with time. The phase angle shift is caused by different oxygen reduction conditions. When the corrosion reaction takes place on the substrate surface, the thinner the electrolyte film is, the faster the mass transportation of oxygen is, and thus the higher the corrosion rate is. The increase in corrosion rate causes the decrease of the time constant, which is reflected by the EIS as a shift of phase angle to the higher frequency.

The phase angle drop could possibly be explained by the dissipation effect. The impedance Z of the capacitor can be described by the following equation:

(3) $$Z=\frac{\omega^{-n}}{Y}\cos(\frac{n\pi }{2})\pm j\frac{\omega^{-n}}{Y}\sin(\frac{n\pi }{2})$$

where Y stands for the admittance, ω stands for the angular frequency of the a. c. voltage, j stands for the unit on imaginary axis, and *n* stands for the dissipation coefficient, which describes the degree of the dissipation effect. During the corrosion process, the change in surface condition results in change in n. Therefore during the first three measures, the variation of *n* causes the drop of phase angle.

**Figure 6.**
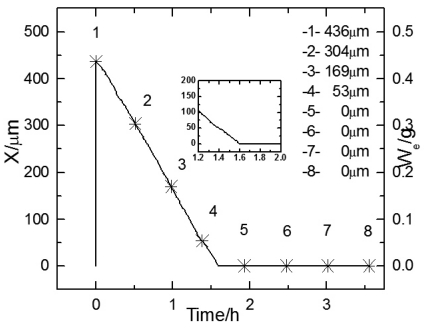
Plots of electrolyte film thickness X *vs.* time during 50^th^ cycle (rusted surface).

Figure 6 shows the change of electrolyte film thickness over time during the 50^th^ wet-dry cycle. The curve is quite similar in shape with that of the 1^st^ cycle, except that the slope of the curve shows a slight change when the curve goes near zero. This is because after the wet-dry cycles, a rust layer with remarkable thickness accumulated on the electrode surface, and hindered the evaporation of the electrolyte film.

**Figure 7.**
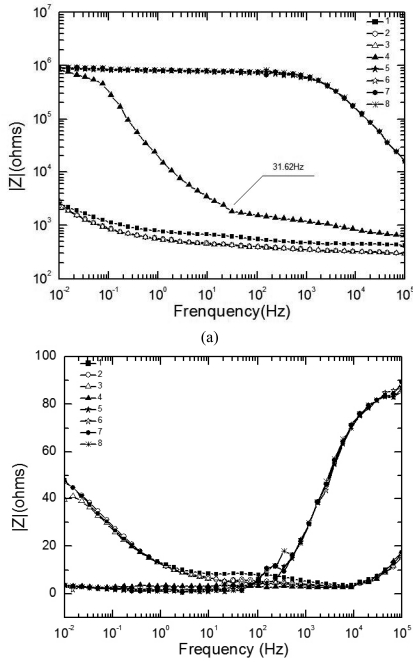
EIS results of 50^th^ cycle: (a) Bode diagram (|Z|); (b)Bode diagram (Phase of Z).

Figures 7a,b display the experimental Bode diagram of all eight measurements in the 50^th^ cycle. The starting time of the 1^st^∼8^th^ EIS measurement in Figure 7 are represented by the data points 1∼8 in Figure 6. It can be seen in Figure 7a that the evolution of EIS also shows an abrupt rise, which began at 31.62 Hz during the fourth measurement. The weight of electrolyte film at this time could be approximated as 0.038 g. This is equivalent to an electrolyte film with a thickness of 38 μm on a substrate surface. However, after the electrode was covered by corrosion product (rust), the electrolyte could be non-homogeneously distributed due to the adsorption of the loose rust. Therefore, it would be inappropriate to describe the electrolyte film on rusted surface by thickness. Minor phase peaks, which show a slight shift, can be observed in the middle of the curve during the first three measurements in Figure 7b.

Comparison of Figures 5 and 7 shows different EIS characteristics between the substrate and rusted surface during both the wet stage and dry stage. In the wet stage, the impedances at high frequency of the solution (R_s_) on a rusted surface are higher than those of the substrate. This can be caused by the hindering effect of the rust layer on the ion migration rate. In the dry stage, the impedance curves of the rusted surface are much lower than those of the substrate. This is because of the fact that for the substrate the electrodes formed an open circuit during the dry stage, while for the rusted surface, the rusts covering the electrode surface connected the two electrodes, and thus decrease the impedance. The phase curves of Z also show obvious differences. Each phase curve of the substrate at wet stage (the first three curves in Figure 5b exhibit only one peak, while each phase curve of rusted surface at wet stage, i.e. the first three curves in Figure 7b exhibits two peaks, one at middle frequency range and one at low frequency range). This is probably caused by different corrosion mechanism of substrate and rusted surface. During the initial stage of corrosion (the substrate), the cathodic reaction is mainly the reduction of oxygen. However, during the later stage, after the rust has been generated as the corrosion product, the reduction of the rust becomes also a part in the cathodic reaction [17].

## 4. Conclusions

From the above work, the following conclusions can be drawn.

1. There exists a critical condition of electrolyte film for corrosion rate of mild steel Q235 both for the substrate and rusted surface during wet-dry cycles.
2. Weight monitoring method can be applied to *in situ* thickness measurement of thin electrolyte films on mild steels. It is also a promising method for the study of other metals with less corrosion resistance. Comb-shaped electrodes are more suitable than electrodes with less electrode area proportions for this method.

## Figures and Tables

**Table 1.** Chemical composition of test material.

**Element**	**C**	**Si**	**Mn**	**P**	**S**	**Cu**
Wt%	0.210	0.210	0.580	0.017	0.036	0.02
